# Protein-Rich Uronic Acid-Containing Polysaccharides from *Juglans regia* Root Bark: Structural Characterization and Structure–Bioactivity Relationships Underlying Multifunctional Biological Activities

**DOI:** 10.3390/polym18141770

**Published:** 2026-07-20

**Authors:** Souha Chokri, Takoua Ben Attia, Asma Haffouz, Basma Hadj Kacem, Sami Mnif, Assad Sila, Ahmed Slaheddine Masmoudi, Ali Ellafi, Sonia Ben Younes

**Affiliations:** 1 Laboratory of Biotechnology and Biogeoresources Valorization, Higher Institute of Biotechnology of Sidi Thabet (ISBST), LR11ES31, Ariana 2020, Tunisia; souhachokri01@gmail.com (S.C.); ahmedsleheddine.masmoudi@isbst.uma.tn (A.S.M.); 2 Department of Life Sciences, Faculty of Sciences of Gafsa, University of Gafsa, Gafsa 2112, Tunisia; assaadsila@gmail.com (A.S.); ali_lafi160@yahoo.fr (A.E.); 3 Laboratory of Population Health, Environmental Aggressors and Alternative Therapies (LR24ES10), Faculty of Medicine of Tunis, University of Tunis El Manar, Tunis 1006, Tunisia; takoua.benattia@fst.utm.tn; 4 Laboratory of Molecular Biotechnology of Eukaryotes, Centre of Biotechnology of Sfax, P.O. Box 1177, Sfax 3018, Tunisia; asmahaffouz@gmail.com (A.H.); basmahadjkacem@gmail.com (B.H.K.); 5 Laboratory of Molecular and Cellular Screening Processes, Centre of Biotechnology of Sfax, P.O. Box 1177, Sfax 3018, Tunisia; sami.mnif@gmail.com; 6 Laboratory for the Improvement of Plants and Valorization of Agroressources, National School of Engineering of Sfax (ENIS), Sfax University, Sfax 3038, Tunisia; 7 Laboratory of Analysis, Treatment and Valorization of Environment Pollutants and Product, Faculty of Pharmacy, Monastir University, Monastir 5000, Tunisia; 8 Biochemistry Department, Faculty of Medicine of Sousse, University of Sousse, Sousse 4002, Tunisia

**Keywords:** protein-rich polysaccharides, uronic acid-containing rich polysaccharides, anionic biopolymers, structure–bioactivity relationships, bioactive polysaccharides, *Juglans regia* root bark

## Abstract

Protein-rich polysaccharides are increasingly recognized as multifunctional biopolymers with significant biomedical potential. In this study, a protein–polysaccharide complex (JrPRP) was isolated for the first time from the root bark of *Juglans regia* L. and comprehensively characterized. JrPRP was obtained with a yield of 4.7% (*w*/*w*) and exhibited an acidic composition enriched in uronic acid-related components, together with minor neutral sugars. Spectroscopic analyses (FTIR and UV–Vis) confirmed the coexistence of carbohydrate and protein domains, while chromatographic profiling (TLC and HPLC) indicated a heterogeneous monosaccharide composition. Scanning electron microscopy revealed a porous and irregular microstructure, consistent with a structured biopolymeric network exhibiting pronounced anionic character. Functionally, JrPRP demonstrated notable antioxidant activity, with IC_50_ values of 405 ± 1.8 µg/mL (DPPH), 225 ± 3.5 µg/mL (ABTS), and 229 ± 1.7 µg/mL (metal chelation), along with strong ferric-reducing capacity. The complex exhibited antibacterial activity against *Pseudomonas aeruginosa*, *Klebsiella pneumoniae*, and *Staphylococcus aureus* (MIC: 2–9 mg/mL), as well as potent antibiofilm activity, inhibiting up to 94% of *Escherichia coli* biofilm formation. Biocompatibility assays indicated low hemolytic activity, supporting its favorable safety profile. In addition, JrPRP showed moderate anticoagulant effects and strong anti-inflammatory activity, reaching 98% inhibition of protein denaturation, comparable to or exceeding diclofenac under similar conditions. These findings identify *J. regia* root bark as a promising and previously underexplored source of structurally distinctive uronic acid-containing protein-rich polysaccharides and provide new insights into the relationship between their compositional features and multifunctional biological activities.

## 1. Introduction

Medicinal plants have historically constituted a fundamental pillar of traditional medicine, providing a rich source of structurally diverse bioactive compounds with therapeutic relevance. In recent decades, scientific interest in aromatic and medicinal plants has intensified due to their expanding pharmaceutical applications in pharmaceuticals, cosmetics, biotechnology, and sustainable agriculture [1]. This renewed attention reflects a global shift toward safer, eco-friendly, and naturally derived agents capable of addressing chronic disorders associated with oxidative stress and inflammation.

*Juglans regia* L. (walnut) is a widely cultivated species of considerable botanical and pharmacological importance, traditionally valued for its diverse therapeutic properties [2]. Its phytochemical profile—comprising polyphenols, flavonoids, tannins, naphthoquinones such as juglone, and essential fatty acids—has been extensively associated with antioxidant, anti-inflammatory, antimicrobial, and cardioprotective activities [2,3,4]. Previous studies have demonstrated the beneficial effects of walnut leaves, seeds, and bark in mitigating cardiovascular and metabolic disorders, as well as certain cancers [3,4,5]. These biological activities are primarily attributed to low-molecular-weight secondary metabolites.

In contrast, the macromolecular carbohydrate fractions of *J. regia*, particularly those derived from root bark tissues, remain largely unexplored. This gap is notable given that root bark represents a metabolically specialized plant compartment enriched in structural and defense-related biopolymers. Since polysaccharide biosynthesis and cell wall composition vary significantly among plant organs, root bark tissues may contain structurally unique carbohydrate assemblies with distinct physicochemical and biological properties.

Plant-derived polysaccharides have emerged as multifunctional macromolecules exhibiting a wide range of biological activities, including antioxidant, antimicrobial, immunomodulatory, and anti-inflammatory effects [6]. Their biodegradability, biocompatibility, low toxicity, and structural diversity make them highly attractive for biomedical, pharmaceutical, and nutraceutical applications [7,8,9]. Importantly, their biological activity is governed not only by monosaccharide composition but also by higher-order structural parameters such as molecular weight, glycosidic linkages, branching degree, and conformational organization, which collectively determine their interactions with biological systems [10].

Among these, protein-rich polysaccharides (or polysaccharide–protein complexes) represent a particularly interesting class of hybrid macromolecules in which carbohydrate chains are associated—either covalently or non-covalently—with protein moieties [11]. This association may enhance functional properties by improving solubility, conformational flexibility, and molecular recognition, thereby influencing structure–bioactivity relationships. Despite their potential, such complexes derived from *J. regia* root bark have not yet been structurally characterized or systematically evaluated.

This knowledge gap is particularly relevant in the context of cardiovascular and thrombotic diseases, which remain leading causes of morbidity and mortality worldwide. Previous studies have demonstrated that walnut bark extracts possess significant antithrombotic properties. For instance, aqueous extracts have been shown to inhibit platelet aggregation induced by ADP, collagen, and thrombin, while also prolonging coagulation times and reducing fibrinogen levels [12,13]. In addition, juglone has been identified as a key compound capable of modulating platelet function through inhibition of Akt phosphorylation and protein disulfide isomerase activity [14]. However, these studies have focused predominantly on low-molecular-weight compounds, leaving the potential contribution of macromolecular fractions largely unexplored.

In parallel, increasing attention has been directed toward acidic polysaccharides, particularly those containing uronic acids, due to their distinctive physicochemical and biological properties. The presence of uronic acid residues introduces negatively charged carboxyl groups into the polysaccharide backbone, conferring a polyanionic character that can influence solubility, molecular conformation, and interactions with proteins, metal ions, and cellular membranes. These features are known to play a key role in modulating antioxidant, anti-inflammatory, and anticoagulant activities [15]. Furthermore, acidic polysaccharides are often part of complex macromolecular assemblies involving protein components, which may enhance their functional properties through synergistic interactions.

Despite the growing interest in plant-derived polysaccharides, the relationship between their structural characteristics and biological activities remains insufficiently understood, particularly for underexplored plant tissues such as root bark. Addressing this gap is also aligned with the principles of sustainable bioeconomy, where underutilized plant materials represent valuable sources of high-added-value biopolymers.

Therefore, the present study aims to (i) extract and characterize protein-rich polysaccharides from *J. regia* root bark, (ii) elucidate their structural and morphological features using complementary analytical techniques including Fourier-transform infrared spectroscopy (FTIR), ultraviolet–visible spectroscopy (UV–Vis), thin-layer chromatography (TLC), high-performance liquid chromatography (HPLC), and scanning electron microscopy (SEM), and (iii) evaluate their multifunctional biological activities, including antioxidant, antibacterial, antibiofilm, hemocompatible, anticoagulant, and anti-inflammatory effects.

By integrating physicochemical characterization with comprehensive biological evaluation, this study establishes a structure–bioactivity framework for protein-rich polysaccharides derived from walnut root bark and highlights their potential as multifunctional biopolymers with promising therapeutic and biotechnological applications.

## 2. Materials and Methods

### 2.1. Chemicals

2,2-Diphenyl-1-picrylhydrazyl (DPPH), 2,2′-azino-bis(3-ethylbenzothiazoline-6-sulfonic acid (ABTS), butylated hydroxytoluene (BHT), potassium ferricyanide, ferric chloride, and crystal violet (c3886) were purchased from Sigma-Aldrich (Darmstadt, Germany).

Saccharide standards, including D-glucose (Glc, CAS: 50-99-7), D-galactose (Gal, CAS: 59-23-4), D-galacturonic acid (GalA, CAS: 91510-62-2), L-rhamnose (Rha, CAS: 10030-85-0), D-mannose (Man, CAS: 3458-28-4), L-arabinose (Ara, CAS: 5328-37-0), and sucrose (Suc, CAS: 57-50-1), were obtained from Sigma Chemical GmbH (Deisenhofen, Germany). Xylose (Xyl, CAS: 7261-26-9) was purchased from the United States Pharmacopeia (Rockville, MD, USA), and glucuronic acid (GlcA, CAS: 6556-12-3) from Fluka Chemika GmbH (Seelze, Germany). These standards were used for TLC and HPLC analyses.

### 2.2. Raw Materials

In this study, *J. regia* root bark was collected in March 2022 from the arid region of Fernana, Tunisia (36°38′59.99″ N, 8°41′59.99″ E) [16]. Botanical identification was performed by Dr. Faouzi Horchani, and a voucher specimen was deposited at the Herbarium of the Faculty of Sciences of Gafsa.

The collected material was thoroughly washed with water to remove surface contaminants, cut into small pieces, and air-dried in the shade at ambient temperature with daily rotation to ensure uniform drying. The dried samples were ground into a fine powder using a laboratory blender (Moulinex) and stored at 4 °C until further analysis [6].

### 2.3. Bacterial Strains

This study involved six bacterial strains: *Staphylococcus aureus* (ATCC 25923), *Pseudomonas aeruginosa* (ATCC 27853), *Klebsiella pneumoniae* (ATCC 13883), *Bacillus cereus* (ATCC 14579), *Escherichia coli* (ATCC 35218), and *Enterobacter* sp. (ATCC 21754). These reference strains were cultured in Mueller–Hinton broth for growth and subsequent testing.

### 2.4. Extraction of Protein-Rich Polysaccharides

**Figure 1:**
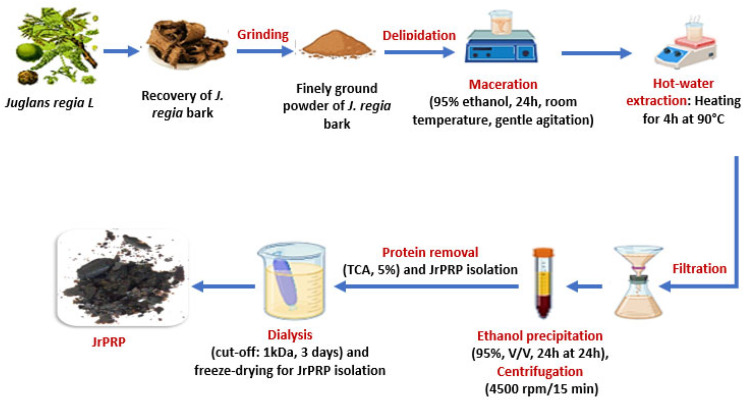
Preparation steps of crude protein-rich polysaccharides from root bark of *J. regia*.

Crude protein-rich polysaccharides were extracted from the root bark of *J. regia* following the procedure previously described [6], with slight modifications. Initially, the delipidated root bark powder was subjected to two extraction cycles using distilled water (solid-to-liquid ratio 1:15, g/mL) at 90 °C for 4 h, followed by filtration. The concentrated sample was precipitated by the addition of 95% (*v*/*v*) ethanol and incubated at 4 °C for 24 h to promote protein-rich polysaccharide precipitation. The resulting mixture was centrifuged at 4500× *g* for 15 min using a refrigerated centrifuge (Jouan KR25i, Thermo Fisher Scientific, Waltham, MA, USA). The collected sample was subsequently treated with 5% (*v*/*v*) trichloroacetic acid (TCA) at room temperature for 2 h under gentle stirring to remove protein released during the extraction process. It is important to note that this treatment was not intended to completely eliminate all proteins. The remaining protein fraction is primarily attributed to structural proteins strongly associated with the polysaccharide matrix, forming a protein-rich polysaccharide complex rather than representing residual impurities. The mixture was then subjected to dialysis (cutoff = 1 kDa) against distilled water for 72 h to eliminate residual salts and low-molecular-weight compounds (Figure 1).

The dialysate was concentrated by rotary evaporation (Heidolph, Germany) and freeze-dried for further analysis. The extraction yield of the protein-rich polysaccharides was calculated according to the method described by Li and Zhang (2009) [17] using the following Formula (1):

Extraction yield (%) = (P_1_/P_0_) × 100 (1)

- P_1_: Weight of the protein-rich polysaccharide extract (g) after evaporation;
- P_0_: Initial weight of the dry sample powder (g).

### 2.5. Spectral Characterization of Protein-Rich Polysaccharide

Protein-rich polysaccharide extracted from the root bark of *J. regia* (JrPRP) was comprehensively analyzed using various spectral, microscopic, and chromatographic techniques.

UV–visible spectroscopy was performed using a Shimadzu UV-1800 PC spectrophotometer (Kyoto, Japan). The crude JrPRP solution was diluted 100-fold, and the absorption spectrum was recorded over a wavelength range of 200–800 nm [18].

Following the extraction of the JrPRP, the solution was evaporated using a rotary evaporator, yielding a powder that was subsequently analyzed. Fourier transform infrared (FTIR) spectrum of JrPRP was recorded using a Shimadzu FT-IR 8400 S spectrophotometer (Kyoto, Japan), spanning the infrared range of 400–4000 cm^−1^ [18].

Scanning electron microscopy (SEM) was used to examine the surface morphology of the JrPRP. The molecules were initially fixed in glutaraldehyde, washed twice with double-distilled water, and dehydrated through an ethanol gradient (25–100%) for 5 min at each concentration. The sample was then dried overnight in a desiccator, mounted on a specimen holder using conductive carbon tape, and sputter-coated with gold via sputter coating. SEM imaging was performed using a field-emission scanning electron microscope (JEOL JSM-IT 100 model, Tokyo, Japan), and images were captured at varying magnifications during the analysis [19].

### 2.6. Biochemical Composition Analysis of the Protein-Rich Polysaccharide

The total and reducing sugar contents of the protein-rich polysaccharide were determined using the phenol–sulfuric acid method and the dinitrosalicylic acid (DNS) assay, respectively. A standard calibration curve was constructed using D-glucose, following established protocols [19,20]. Total sugar content was quantified spectrophotometrically at 490 nm. Protein concentration was measured using the Bradford assay (Bio-Rad Laboratories, Hercules, CA, USA), with bovine serum albumin (BSA) as the reference standard [21]. Crude fat content was assessed gravimetrically by Soxhlet extraction of dried samples using hexane as the solvent [22]. All assays were conducted in triplicate to ensure accuracy and reproducibility.

### 2.7. Determination of Monosaccharide Composition

The monosaccharide composition of the JrPRP was analyzed using a hydrolysis approach. Briefly, 5 mg of JrPRP samples from the root bark of *J. regia* was hydrolyzed in trifluoroacetic acid (TFA, 4 M) in sealed tubes at 100 °C for 2, 4, and 6 h. After hydrolysis, the samples were cooled to room temperature, and TFA was co-evaporated with methanol under vacuum to remove excess acid. This purification step was repeated two to three times with methanol to ensure complete removal of impurities [6]. The hydrolyzed JrPRP sample, along with standard sugars (D-glucose (Glc), D-galactose (Gal), D-galacturonic acid (GalA), L-rhamnose (Rha), D-mannose (Man), L-arabinose (Ara), sucrose (Suc), xylose (Xyl), and glucuronic acid (GlcA)), was then analyzed by thin-layer chromatography (TLC) on a silica gel plate (Merck, Darmstadt, Germany). The separation was performed using a mobile phase composed of chloroform, acetic acid, and water (6:7:1, *v*/*v*/*v*). After drying the plate with warm air, it was sprayed with a mixture of 95% ethanol and 5% H_2_SO_4_ and then heated at 105 °C for 10 min to develop colored spots. The retention factor (Rf) was calculated as the ratio of the distance traveled by the sugar to the distance traveled by the mobile phase during TLC analysis [6].

### 2.8. High-Performance Liquid Chromatography (HPLC) Conditions

Chromatographic analysis was conducted using an Agilent 1100 series system equipped with a quaternary pump, a refractive index detector (RID), manual injector, and data acquisition and processing software (LC-Solution, chem32). The hydrolyzed JrPRP was analyzed using a C_18_ column (Aminex HPX-87C, 250 × 4.0 mm) optimized for sugar analysis. A 20 µL sample injection volume was used. Demineralized water served as the mobile phase, facilitating the separation of the sugar monomers at a constant flow rate of 0.6 mL/min.

### 2.9. Biological Properties of Protein-Rich Polysaccharide Extracted from the Root Bark of J. regia

#### 2.9.1. In Vitro Antioxidant Activities of Protein-Rich Polysaccharide from *J. regia* Root Bark

The antioxidant activity of JrPRP was thoroughly examined using a series of in vitro assays.

The DPPH radical scavenging activity was evaluated according to the method outlined by Zhou et al. (2023) [19]. In this assay, the reduction of the DPPH radical was quantified by spectrophotometrically measuring the decrease in absorbance at 517 nm after a 30 min incubation period in the dark at room temperature. The radical scavenging activity (RSA) was calculated using the Formula (2):

% RSA = [(A_DPPH_ − A_E_)/A_DPPH_] × 100 (2)

- RSA: Radical scavenging activity;
- A_E_: Absorbance of the antioxidant solution;
- A_DPPH_: Absorbance of the DPPH solution.

The ABTS free radical-scavenging activity was subsequently assessed following the protocol outlined by Zhou et al. (2023) [19]. In this assay, the ability of the protein-rich polysaccharide to quench ABTS radical cations was evaluated, and the percentage inhibition of ABTS^+^ radicals was determined by measuring the absorbance at 734 nm using a Shimadzu UV-mini 1240 spectrophotometer (Shimadzu Corporation, Kyoto, Japan). The percentage ABTS^+^ radical inhibition was calculated using the following Formula (3):

ABTS radical scavenging activity (%) = [(1 − (A/A_0_)) × 100] (3)

- A_0_: Absorbance of the control;
- A: Absorbance of the sample.

The reducing power of JrPRP was evaluated based on its capacity to reduce ferric ions, as described by Zhou et al. (2023) [19]. A blank solution, prepared with water instead of the sample, was used, and the absorbance of the reaction mixture was measured at 700 nm after 10 min of incubation in the dark. This assay gauges the formation of ferric iron ion complex, which serves as an indicator of the reducing potential of JrPRP.

The ferrous ion (Fe^2+^) chelation ability of JrPRP was assessed following the method proposed by Xu et al. (2021) [23]. The ability of JrPRP to bind and sequester ferrous ions was determined by measuring the decrease in the absorbance at 562 nm. The results were expressed as the IC_50_ value, and the percentage inhibition of ferrozine-Fe^2+^ complex formation was calculated using the following Formula (4):

Inhibition (%) = 100 × ((A − (B − C)/A)(4)

- A: Absorbance of the control: the control contained FeCl_2_ and ferrozine without JrPRP;
- B: Absorbance without FeCl_2_;
- C: Absorbance with FeCl_2_.

#### 2.9.2. Antibacterial Activities: Minimum Inhibitory Concentration (MIC) and Minimum Bactericidal Concentration (MBC)

The MIC and MBC of JrPRP were determined using standardized protocols [6]. The broth dilution method was used with the same microbial reference strains. Each dilution was incubated with an exponential phase bacterial inoculum (10^6^ CFU/mL) in the wells of an enzyme-linked immunosorbent assay (ELISA) plate. The MIC was ascertained through serial dilution of the JrPRP solution in Mueller–Hinton broth, with each well containing 10 μL of broth and 10 μL of bacterial inoculum. The initial bacterial concentration was adjusted to 10^6^ CFU/mL, and the tested concentrations of JrPRP ranged from 0 to 512 μg/mL. Control wells were included for sterility (negative control, no inoculum) and inoculum viability (positive control, no extract). After 24 h of incubation at 37 °C, bacterial growth was assessed by measuring the turbidity and pellet formation in the wells. The MIC was defined as the lowest concentration at which no visible bacterial growth occurred during the 24 h incubation period. All tests were performed in triplicate.

To determine the MBC, 10 μL of medium was extracted from the wells that exhibited no visible growth and plated onto Mueller–Hinton agar. After an additional 24 h of incubation at 37 °C, the number of viable organisms was counted as CFU/mL. MBC was defined as the lowest concentration that resulted in a ≥99% reduction in bacterial viability. Each experiment was performed at least twice.

The MIC and MBC values obtained were specific for the extracts of each strain tested. The extract was classified as bactericidal if the MBC/MIC ratio was 1:2 and bacteriostatic if the ratio was ≥2 [6].

#### 2.9.3. Antibiofilm Activity

The antibiofilm activity of the protein-rich polysaccharide was evaluated using the serial dilution method in 96-well flat-bottom microplates (Nunc, Roskilde, Denmark) [24]. Pathogenic strains were cultured overnight in Tryptic Soy Broth (TSB) (Difco, Franklin Lakes, NJ, USA) at 37 °C and then diluted (1:100) in fresh TSB supplemented with 2% glucose to obtain a final bacterial concentration of 10^6^ CFU/mL. For each well, 100 μL of the bacterial suspension was mixed with 100 μL of JrPRP prepared in TSB at concentrations ranging from 6.2% to 75% (*v*/*v*), resulting in final JrPRP concentrations of 3.1% to 37.5% (*v*/*v*) in the wells. Control wells consisted of TSB supplemented with glucose and JrPRP without bacteria (negative control), and TSB supplemented with glucose and bacterial strains without JrPRP (positive control). The plates were incubated at 37 °C for 24 h to allow biofilm formation.

Following incubation, unattached cells were removed by washing each well twice with 200 μL sterile phosphate-buffered saline (PBS, 7 mM Na_2_HPO_4_, 3 mM NaH_2_PO_4_, and 130 mM NaCl, pH 7.4). The plates were air-dried and stained with 150 μL of 1% crystal violet (Merck, France) solution in 20% ethanol for 15 min at room temperature. Excess stain was removed by rinsing the wells thrice with sterile water. Subsequently, 200 μL of 30% (*v*/*v*) glacial acetic acid was added to each well, and the plates were incubated for 1 h at room temperature. Optical density (OD_570_) was measured using a microplate reader (Multiscan FC, Thermo Fisher Scientific).

All tests were performed in triplicate, and the biofilm inhibition concentration (BIC) was calculated using the following Formula (5):

BIC (%) = ((OD_Control_ − OD_Sample_)/OD_Control_) × 100 (5)

#### 2.9.4. Hemolysis Assay

##### Blood Agar Hemolysis Assay

The hemolytic potential of JrPRP was further evaluated qualitatively using 5% sheep blood agar plates. Wells were loaded with 100 μL of JrPRP at various concentrations (30–120 mg/mL) and incubated at 37 °C for 24–48 h [25].

##### Cytotoxicity Assessment on Human Erythrocytes

Human erythrocytes were separated by centrifugation at 3000 rpm, washed twice with phosphate buffer (pH 7.40), and resuspended in NaCl solution. The cells were incubated for 15 min at 37 °C, followed by the addition of 20 µL of JrPRP (300 mg/mL) and a further 15 min incubation. Blood smears were then prepared, air-dried, and stained using a modified May–Grünwald–Giemsa method [25]. Briefly, the slides were first stained with May–Grünwald solution for 3 min without rinsing. Following this, 0.1 M phosphate buffer (pH 7.4) was added, and once a metallic sheen appeared, the slides were gently rinsed with distilled water. The slides were then counterstained with Giemsa in buffered solution for 20 min, washed, and air-dried before microscopic examination.

Microscopic evaluation of the erythrocytes was conducted to assess any morphological changes. The appearance of echinocytes (spiky cells) indicated cytotoxicity, while the presence of intact cells suggested biocompatibility. A positive control with H_2_O_2_ was used to induce echinocyte formation, confirming the sensitivity of the assay [26].

##### Membrane Stabilization Against Hypotonicity-Induced Hemolysis

The membrane protective effect of JrPRP was evaluated based on the method by Abbou et al. [27]. A hypotonic phosphate buffer (PBS, 10 mM, 50 mM NaCl, pH 7.4, 5 mL) containing JrPRP at different concentrations (30–300 mg/mL) was mixed with 0.50 mL of erythrocyte suspension. After incubation at 37 °C for 10 min, the mixtures were centrifuged at 3000 rpm for 10 min. The absorbance of the supernatant was measured at 540 nm to determine the extent of hemolysis. The experiments were repeated three times for reproducibility. The percentage of hemolysis inhibition was calculated using the following Formula (6):

Inhibition (%) = 100 × (A_0_ − A_1_)/A_0_ (6)

where A_0_ represents the absorbance of the positive control (hypotonic buffer without JrPRP), and A_1_ is the absorbance with the test sample. The negative control consisted of erythrocytes incubated with isotonic PBS.

#### 2.9.5. Coagulation and Platelet Function Assays

All experimental procedures involving blood or plasma were performed with samples collected from healthy, drug-free adult volunteers aged between 25 and 40. The study protocol was reviewed and approved by the Ethics Committee of the Blood Transfusion Center of Sfax, Tunisia. Written informed consent was obtained from all donors in accordance with the ethical principles of the revised Declaration of Helsinki (October 2013). All assays were conducted in triplicate to ensure the accuracy and reproducibility of results.

##### Anticoagulant Activity

Activated Partial Thromboplastin Time (APTT)—Intrinsic Pathway

To assess the impact of JrPRP on the intrinsic coagulation pathway, 50 μL of human plasma was incubated with 10 μL of JrPRP solution (25, 50, and 100 μg/mL) and 50 μL of APTT reagent at 37 °C for 3 min. Coagulation was initiated by adding 50 μL of 25 mM CaCl_2_, and the clotting time was recorded using a coagulometer. Heparin was used as the positive control [28].

Prothrombin Time (PT) and International Normalized Ratio (INR)—Extrinsic Pathway

Similarly, 50 μL of plasma were mixed with 10 μL of JrPRP solution (25, 50, and 100 μg/mL) and incubated at 37 °C for 3 min. The coagulation cascade was triggered by adding 50 μL of thromboplastin reagent, and the clotting time (prothrombin time, PT, in seconds) was recorded using a coagulometer. Heparin served as a reference anticoagulant [28].

The obtained PT values were further used to calculate the International Normalized Ratio (INR), which standardizes prothrombin time measurements according to the following Equation (7):

INR = (PT_sample_/PT_normal mean_)^ISI^ (7)

where PT_sample_ is the clotting time of the tested sample, PT_normal mean_ corresponds to the mean clotting time of normal plasma, and ISI represents the International Sensitivity Index to the thromboplastin reagent. INR values were determined according to standard conversion procedures described in the literature. The International Sensitivity Index (ISI) was not explicitly provided for the reagent lot used in this study. Therefore, based on manufacturer data and the published literature for Neoplastin reagents used on Stago analyzers, an approximate ISI value of 1.3 was considered [29].

##### Platelet Preparation and Aggregation Assay

Platelet-Rich Plasma (PRP) and Platelet-Poor Plasma (PPP) were prepared by centrifugation of collected blood. PRP was incubated with various concentrations of JrPRP or 1% Triton (positive control) in 96-well ELISA plates. Acid phosphatase activity, indicative of platelet viability, was assessed by adding 5 mM *p*-nitrophenyl phosphate and incubating at 37 °C for 1 h. The enzymatic reaction was initiated by adding 5 mM *p*-nitrophenyl phosphate and incubating at 37 °C for 1 h. The reaction was terminated by adding 2N NaOH, and absorbance was measured at 405 nm. Viability was expressed as a percentage, with untreated platelets representing 100% viability and Triton-treated cells representing 0% [30].

For the platelet aggregation assay, PRP samples preincubated with JrPRP or ethanol were stimulated with ADP, collagen, or arachidonic acid. Aggregation was monitored for 15 min using a Chrono-Log aggregometer. Experiments were performed using blood samples from at least five individual donors to ensure robustness and reproducibility [30].

#### 2.9.6. Cytotoxicity Evaluation Using the MTT Assay

To assess the biocompatibility of JrPRP, its cytotoxicity was investigated in vitro using the MTT assay on HEK-293 cells, a widely accepted model for non-tumorigenic human cells in toxicological research. This cell line, obtained from the American Type Culture Collection (ATCC), provides a reliable platform to evaluate the safety of novel bioactive compounds.

HEK-293 cells were cultured in RPMI-1640 medium supplemented with 10% fetal bovine serum (FBS), 1% L-glutamine, and gentamicin. Cells were maintained at 37 °C in a humidified incubator with 5% CO_2_. For cytotoxicity testing, cells were seeded in 96-well flat-bottom plates at a density of 8 10^4^ cells/well and allowed to adhere for 24 h. They were then exposed to a range of JrPRP concentrations for an additional 24 h. Untreated wells served as the negative control.

Following incubation, cell viability was determined using the MTT reagent (0.5 mg/mL), which was added to each well and incubated for 4 h. The resulting formazan crystals, formed by mitochondrial enzymatic reduction, were solubilized in DMSO, and the absorbance was measured at 570 nm using a Flash Varioskan microplate reader (Thermo Scientific, Waltham, MA, USA). All experiments were conducted in triplicate to ensure reproducibility. Cell viability was expressed as a percentage relative to the control group, using the following Formula (8) [30]:

Cell Viability (%) = [(AE − AC)/AC] × 100(8)

where AE is the absorbance of the treated cells, and AC is the absorbance of the untreated control.

#### 2.9.7. In Vitro Evaluation of Anti-Inflammatory Activity

##### In Vitro Evaluation of Anti-Inflammatory Activity Using BSA Denaturation Assay

The anti-inflammatory potential of JrPRP was investigated through its capacity to inhibit heat-induced denaturation of bovine serum albumin (BSA) [31]. Samples of JrPRP, at concentrations ranging from 10 to 400 μg/mL, were prepared along with standard solutions of diclofenac used for comparison. Each sample was mixed with 0.05 mL of a 0.5% (*w*/*v*) BSA solution and incubated at 37 °C for 20 min. The mixtures were then exposed to 57 °C for 3 min before being cooled to room temperature. Following this, 2.5 mL of phosphate buffer was added to each tube.

Absorbance was recorded at 255 nm using a UV–visible spectrophotometer to evaluate the extent of protein denaturation. The control sample (lacking any JrPRP or standard drug) was considered to represent 100% protein denaturation. The inhibitory effect of JrPRP on protein denaturation was calculated using the following Formula (9):

Inhibition (%) = [(OD_control_ − OD_sample_)/OD_control_] × 100 (9)

OD_control_ indicates the absorbance of the control of JrPRP sample, while OD_sample_ refers to the absorbance of the test. The findings represent the average of three replicates.

##### In Vitro Evaluation of Anti-Inflammatory Activity Using Egg Albumin Denaturation Assay

A complementary assay was conducted to assess the ability of JrPRP to prevent denaturation of egg albumin [31]. A 5 mL solution was prepared by combining 0.20 mL of egg albumin, 2.80 mL of phosphate-buffered saline (PBS, pH 6.40), and 2 mL of JrPRP at concentrations of 1, 10, 100, and 150 µg/mL. Diclofenac was used as a reference drug at concentrations of 10 to 200 µg/mL. Diclofenac at the same concentrations served as the reference drug. A control solution containing distilled water was used as a negative control. The prepared mixtures were incubated at 37 ± 2 °C for 15 min and then heated to 70 °C for 5 min. After cooling, the absorbance was measured at 660 nm. The percentage inhibition of protein denaturation was determined using the Formula (10):

Inhibition (%) = [(OD_control_ − OD_sample_)/OD_sample_] × 100 (10)

OD_sample_ indicates the absorbance of JrPRP sample, while OD_control_ refers to the absorbance of the control. The findings represent the average of three replicates.

### 2.10. Statistical Analyses

The results were analyzed using Statistical Package for the Social Sciences (IBM, SPSS) software version 26.0. All values are expressed as the means ± standard deviation (SD) from triplicate experiments.

## 3. Results and Discussion

### 3.1. Extraction Yield and Recovery of Protein-Rich Polysaccharides from J. regia Root Bark

Protein-rich polysaccharides were extracted from *J. regia* root bark using a hot water extraction method followed by ethanol precipitation, deproteinization, and dialysis. Hot water extraction was selected for its ability to preserve the native conformation of polysaccharide–protein complexes while avoiding structural alterations associated with harsh chemical treatments [19]. This approach is particularly suitable for maintaining biologically relevant functional groups, including uronic acid residues and protein-associated moieties, which are likely critical for the observed bioactivities [32].

The purified JrPRP fraction was obtained as a homogeneous pale-brown powder, indicating effective removal of low-molecular-weight pigments and interfering secondary metabolites. The overall extraction yield reached 4.7 ± 1.1% (*w*/*w*) based on dry root bark.

This yield is comparable to, and in some cases slightly higher than, those reported for polysaccharides isolated from other parts of *J. regia* and related plant matrices. For instance, polysaccharides extracted from walnut shells under optimized hot water conditions yielded approximately 3.5% [33]. The relatively higher recovery from root bark may reflect organ-specific enrichment in structural or storage polysaccharides, likely associated with differences in cell wall composition and carbohydrate metabolism between subterranean and aerial tissues.

More broadly, the obtained yield falls within the typical range reported for plant-derived polysaccharides (generally 1–10%), including those from *Cistanche deserticola*, *Schinus molle*, *Rhamnus alaternus*, and various *Astragalus* species [6,17,34,35,36,37,38,39]. However, beyond quantitative comparison, extraction yield alone does not necessarily reflect functional quality, as it depends on both the intrinsic polysaccharide content of the plant material and the efficiency of the extraction process in preserving structural integrity.

The recovery of nearly 5% protein-rich polysaccharides suggests that *J. regia* root bark represents a meaningful reservoir of macromolecular biopolymers. Considering the structural and protective role of root bark in plants, it is reasonable to hypothesize that its polysaccharide fraction may exhibit distinctive structural features, such as elevated uronic acid content, specific branching patterns, or stronger protein association. These characteristics may influence physicochemical properties and biological functions, particularly in processes involving redox regulation, microbial interactions, and coagulation pathways [15].

Overall, the extraction yield obtained in this study not only confirms the feasibility of isolating protein-rich polysaccharides from *J. regia* root bark but also supports its valorization as a renewable source of multifunctional biopolymers. Subsequent structural and functional analyses were therefore undertaken to elucidate the relationship between the molecular architecture of JrPRP and its biological activities.

### 3.2. Biochemical and Structural Characterization of JrPRP

#### 3.2.1. Biochemical Composition and Monosaccharide Profile

##### Global Biochemical Composition

The biochemical composition of JrPRP revealed a carbohydrate-dominant yet protein-rich macromolecular system, comprising 63.2 ± 0.4% total sugars, 40.3 ± 0.1% reducing sugars, 34.1 ± 0.1% proteins, and 2.6 ± 0.2% lipids (Table 1). This composition indicates that the extracted fraction corresponds to a protein-associated polysaccharide complex rather than a purified neutral polysaccharide.

The relatively high proportion of reducing sugars suggests the presence of accessible terminal residues or partially hydrolyzed/branched regions within the polysaccharide chains, which may contribute to enhanced redox reactivity and interaction with biological targets. In addition, the substantial protein content (~34%) supports the presence of polysaccharide–protein assemblies, likely stabilized through covalent linkages and/or strong non-covalent interactions. Such macromolecular complexes are increasingly reported to exhibit enhanced biological properties compared to isolated polysaccharides, particularly in antimicrobial and immunomodulatory applications.

The low lipid content confirms the efficient removal of hydrophobic constituents during purification and highlights the predominance of hydrophilic polymeric components. From a structural perspective, the coexistence of carbohydrate and protein fractions suggests a composite architecture capable of forming supramolecular networks via hydrogen bonding and electrostatic interactions. This organization may influence solubility, conformational flexibility, and the accessibility of reactive functional groups, including hydroxyl and carboxyl moieties.

Collectively, this biochemical profile identifies JrPRP as a structurally complex, multifunctional, protein-rich acidic biopolymer enriched in both saccharide and protein domains, thereby warranting further investigation into its detailed structural features and their relationship with the observed biological activities.

##### Monosaccharide Composition Determined by TLC and HPLC

Qualitative thin-layer chromatography (TLC) analysis of acid-hydrolyzed JrPRP, monitored at 2, 4, and 6 h, revealed the presence of glucose, galactose, arabinose, and glucuronic acid, based on retention factor (Rf) values consistent with authentic standards. A transient sucrose spot (Rf = 0.2) was detected at the early hydrolysis stage (2 h) but disappeared upon prolonged acid treatment, indicating its progressive hydrolytic cleavage into constituent monosaccharides.

The migration behavior of sugars on TLC plates reflected their polarity and structural characteristics. Neutral monosaccharides such as glucose and galactose, rich in hydroxyl groups, exhibited moderate mobility, whereas the more polar uronic acid showed reduced migration due to stronger hydrogen bonding interactions with the silica stationary phase [6]. These observations are consistent with previous TLC analyses of *Rhamnus alaternus* polysaccharides, where stem extracts contained glucose, galactose, xylose, arabinose, and glucuronic acid, while leaf extracts included rhamnose and galacturonic acid, reflecting distinct monosaccharide profiles associated with tissue-specific biosynthetic patterns [6]. Similar compositions have also been described for polysaccharides from *Pistacia vera* shells and *Schinus molle* and *S. terebinthifolius*, which include rhamnose, glucose, galactose, mannose, xylose, arabinose, and galacturonic acid [9,37].

**Figure 2:**
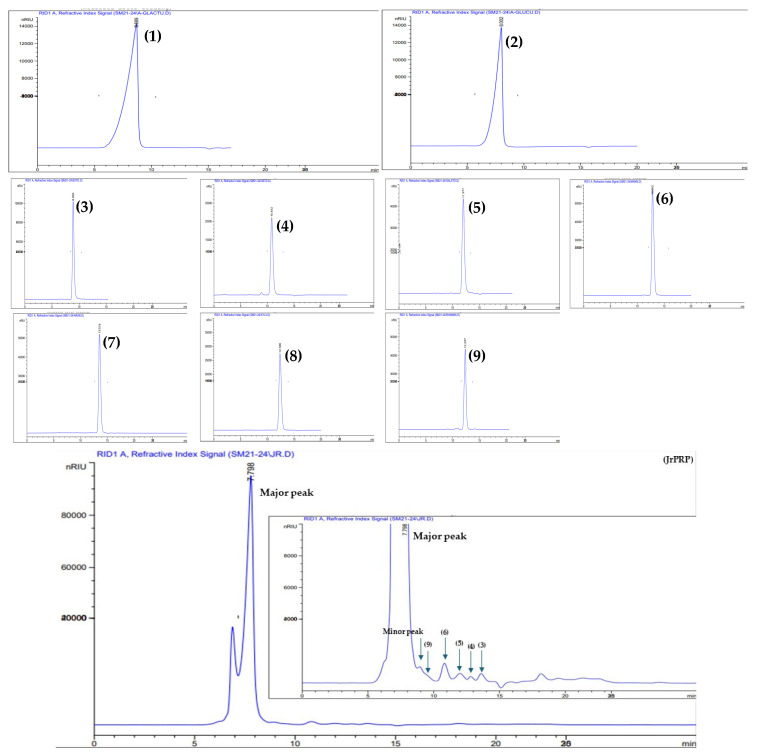
HPLC chromatograms of monosaccharide standards and the acid-hydrolyzed JrPRP fraction recorded at 245 nm. Separation was performed using a C18 Aminex HPX-87C column (250 × 4 mm) with demineralized water as the mobile phase under isocratic conditions (flow rate: 0.6 mL min^−1^, 40 min). The upper panels correspond to individual monosaccharide standards, including glucuronic acid (1), galacturonic acid (2), glucose (3), galactose (4), arabinose (5), mannose (6), xylose (7), rhamnose (8), and sucrose (9), used for retention time comparison. The lower chromatogram represents the hydrolyzed JrPRP sample, showing a dominant peak along with minor signals corresponding to neutral sugars. Retention times of standards were used to tentatively assign peaks in the JrPRP sample chromatogram.

Quantitative high-performance liquid chromatography (HPLC) analysis of the hydrolyzed fraction (Figure 2) further elucidated the monosaccharide composition of JrPRP. The chromatogram was dominated by a major peak at a retention time (RT) of 7.798 min, representing 92.2 ± 1.2% of the total chromatographic area, along with a minor peak at 8.924 min and several low-abundance components. This dominant fraction may correspond to a modified uronic acid derivative or a conjugated carbohydrate structure, requiring further LC–MS or NMR confirmation.

Comparison with reference standards—including glucuronic acid (RT = 8.002 min), galacturonic acid (RT = 8.689 min), glucose (RT = 10.832 min), galactose (RT = 11.977 min), arabinose (RT = 13.578 min), mannose (RT = 12.862 min), xylose (RT = 12.369 min), rhamnose (RT = 12.297 min), and sucrose (RT = 8.894 min)—suggests that the minor peak at 8.924 min is close to the retention time of galacturonic acid, suggesting a possible correspondence with galacturonic acid. Additional minor signals were detected corresponding to common neutral sugars, including a transient sucrose-related peak (~2.1%) at early hydrolysis stages and small contributions from glucose (2.1%), galactose (1.5%), arabinose (1.3%), and mannose (0.7%).

Comparable heteropolysaccharide compositions have been reported for acidic fractions from *Brassica rapa* L. rhizomes, where fractions such as BRP-1, BRP-2, and BRP-3-1 were found to comprise mannose (Man), rhamnose (Rha), galacturonic acid (GalA), glucose (Glc), galactose (Gal), and arabinose (Ara), indicating their heteropolysaccharide nature [40].

Notably, the dominant peak, representing more than 90% of the total chromatographic area, does not exactly match the retention times of any analyzed standards, including glucuronic acid. This discrepancy may arise from hydrolysis-induced transformations, matrix effects, or the presence of structurally complex or conjugated carbohydrate forms associated with the protein-rich nature of the sample. Accordingly, peak assignment based solely on retention time should be interpreted with caution.

Despite these limitations, the predominance of a single chromatographic signal suggests that JrPRP is largely composed of a major carbohydrate-derived fraction. This conclusion is supported by complementary FT-IR analysis, which revealed characteristic absorption bands of carboxylate groups, TLC confirming carbohydrate presence, and UV–visible spectroscopy indicating polysaccharide–protein complexes.

Collectively, these findings provide converging evidence that JrPRP contains uronic acid residues and exhibits an overall acidic and anionic character. The presence of carboxyl functional groups likely contributes to key physicochemical properties, including aqueous solubility, metal-chelating capacity, and interactions with positively charged biomolecules such as coagulation factors, membrane proteins, and microbial surfaces. From a structure–function perspective, this acidic and anionic profile offers a plausible molecular basis for several biological activities reported in this study, including antioxidant, antimicrobial, and antibiofilm effects. These properties may arise from electrostatic interactions, metal ion chelation, and interference with microbial adhesion processes.

Given the limited number of studies specifically addressing *Juglans regia* polysaccharides, comparisons with structurally related plant-derived polysaccharides were undertaken to contextualize the compositional features of JrPRP. In contrast to other systems, JrPRP displays a distinct profile dominated by a single unresolved carbohydrate fraction with minor contributions from neutral sugars. For example, polysaccharides from *Schinus molle* and *S. terebinthifolius* fruits are rich in arabinose (40.5–42.1%) and galacturonic acid (31.2–41.1%) [37], whereas *Pistacia vera* shell polysaccharides exhibit a more diverse monosaccharide composition, including rhamnose, glucose, galactose, mannose, xylose, arabinose, and galacturonic acid [9]. Similarly, *Polygonatum cyrtonema* polysaccharides show a balanced distribution of monosaccharides, with neutral sugars such as mannose (2.2%), glucose (2.4%), galactose (2.4%), xylose (2.3%), ribose (2.3%), fucose (1.8%), rhamnose (2.5%), arabinose (2.3%), and uronic acids (galacturonic (2.5%), and glucuronic (2.2%) acids) each representing approximately 2–3% of total composition [41]. In another study, an acidic polysaccharide (BRP-3-1) from *Brassica rapa* L. rhizomes consisted predominantly of galacturonic acid (75.584%), along with rhamnose (1.687%), galactose (14.452%), and arabinose (8.277%), confirming its heteropolysaccharide nature [40].

These comparisons highlight the structural complexity and potential uniqueness of JrPRP, which may reflect organ-specific biosynthetic pathways in *Juglans regia* root bark. It is important to emphasize that JrPRP represents a crude, protein-rich fraction rather than a fully purified polysaccharide. Therefore, although current chromatographic and spectroscopic evidence supports the presence of an acidic polysaccharide–protein complex, further structural elucidation—particularly using LC–MS, NMR spectroscopy, and molecular weight distribution analysis—is necessary to fully define its molecular architecture.

#### 3.2.2. Spectroscopic and Microscopic Characterization

##### Ultraviolet–Visible Spectroscopy

**Figure 3:**
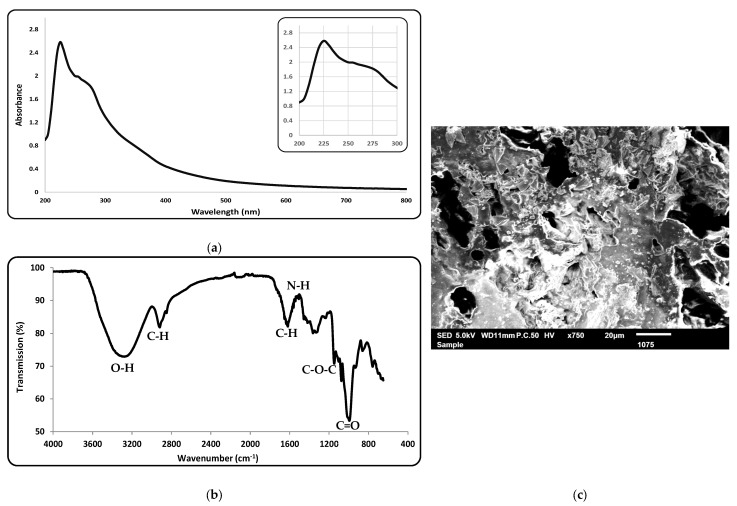
(**a**) UV–visible, (**b**) FT-IR spectra and (**c**) SEM images of the surface structures obtained at a magnification of 5 K (20 μm) of protein-rich polysaccharides from *J. regia*.

The UV–Vis spectrum of JrPRP (Figure 3a) exhibited distinct absorption bands at approximately 225, 255, and 275 nm. The strong absorption observed around 225 nm can be attributed to π→π* electronic transitions associated with carbonyl and carboxyl groups, which are characteristic of polysaccharides containing uronic acid residues.

The absorption bands detected at 255 and 275 nm indicate the presence of protein components, likely arising from aromatic amino acids such as tyrosine and tryptophan. These observations are consistent with the relatively high protein content determined in the compositional analysis and support the presence of a polysaccharide–protein complex. The absence of a pronounced absorption peak at 260 nm suggests minimal contamination by nucleic acids, thereby confirming the effectiveness of the purification procedure.

In contrast, previously reported purified polysaccharides from *J. regia* shells exhibited no characteristic absorption peaks at 260 nm (nucleic acids), 280 nm (proteins), or 620 nm (pigments) within the 200–800 nm spectral range, reflecting their high purity and lack of proteinaceous constituents [33]. This marked difference in spectral behavior highlights the distinctive protein-associated nature of JrPRP compared with fully purified polysaccharide fractions derived from other plant organs.

Overall, the UV–Vis spectral features of JrPRP corroborate the presence of an acidic, uronic acid–dominated polysaccharide matrix structurally associated with protein residues. The dominance of π → π* transitions in the low-wavelength UV region further supports the strong anionic character of this macromolecule, which likely underlies its capacity for electrostatic interactions and contributes to its observed biological activities. These findings are in full agreement with the compositional and structural analyses and reinforce the hybrid polysaccharide–protein architecture of this fraction.

Fourier Transform Infrared Spectroscopy

Fourier Transform Infrared (FT-IR) spectroscopy was employed to elucidate the functional group architecture of JrPRP. The FT-IR spectrum (Figure 3b) displayed a broad and intense absorption band in the 3500 and 3200 cm^−1^ region, corresponding to O–H and N–H stretching vibrations. This feature reflects extensive hydrogen bonding within the carbohydrate–protein network and confirms the polysaccharide nature of the sample [42]. Characteristic aliphatic C–H stretching bands were observed at 2914 cm^−1^ and 2847 cm^−1^, corresponding to methylene (–CH_2_) and methyl (–CH_3_) groups, respectively, which are typical of carbohydrate structures.

A prominent absorption band at 1617 cm^−1^ was assigned to overlapping contributions from amide-related vibrations (C=O stretching of amide I, N–H bending of amide II, and C–N stretching), thereby supporting the presence of protein residues. Together with bands at 1559 cm^−1^ and 1418 cm^−1^, these signals are also associated with the asymmetric and symmetric stretching vibrations of carboxylate groups (COO^−^), which are characteristic of uronic acids [6,43,44,45]. The overlap between amide and carboxylate vibrations likely contributes to reduced band resolution and lower apparent intensity of the carboxylate signals. The relative intensity of these bands is consistent with the high proportion of uronic acid-related components suggested by HPLC analysis. An additional band near 950 cm^−1^ further corroborates the presence of uronic acid residues [44].

Although weak amide I (≈1650 cm^−1^) and amide II (≈1540 cm^−1^) bands were detected, their relatively low intensity suggests that proteins are not organized into well-defined secondary structures but rather exist in a disordered state or are strongly associated with the polysaccharide matrix. This interpretation is consistent with the Bradford assay, which indicated a residual protein content of approximately 34% (*w*/*w*), suggesting that proteins are likely present as polysaccharide-bound or glycosylated components, with their spectral contributions partially masked by dominant carbohydrate signals.

The relatively high protein content supports the formation of polysaccharide–protein complexes rather than simple physical mixtures. Such macromolecular assemblies are increasingly recognized for exhibiting enhanced biological performance compared with purified polysaccharides alone, as protein domains may facilitate molecular recognition, improve aqueous solubility, and promote interactions with biological membranes.

In the fingerprint region (1200–950 cm^−1^), characteristic polysaccharide bands corresponding to C–C, C–O–H, and C–O–C stretching vibrations of pyranose rings were clearly observed [46]. Peaks at 1020, 1050, and 984 cm^−1^ are consistent with typical carbohydrate spectral profiles [46]. This region, particularly between 1170 and 980 cm^−1^, is sensitive to the axial or equatorial orientation of hydroxyl groups in monosaccharides, allowing discrimination of sugar configurations such as xylose, glucose, and galactose in pyranose forms based on vibrational contributions of hydroxyl groups and neighboring carbons at positions C2, C3, and C4 [47,48]. In JrPRP, the band at 1091 cm^−1^ corresponds to C–OH stretching vibrations, while the absorption between 1150 and 1160 cm^−1^ is attributed to glycosidic C–O–C stretching involving the anomeric carbon.

A weak band around 1075 cm^−1^ was also observed; however, its assignment to sulfur-containing groups such as sulfoxide (S=O) should be interpreted with caution, as this region significantly overlaps with C–O stretching vibrations typical of polysaccharides. Moreover, no sulfur-containing reagents were used during the extraction process, and no complementary elemental analyses were performed to confirm the presence of sulfur. Therefore, this band is more reliably attributed to polysaccharide-related vibrations, and no definitive FTIR evidence of sulfation can be established. Overall, the FT-IR spectral profile strongly supports the presence of an acidic, uronic acid-rich polysaccharide framework structurally associated with protein components, thereby confirming the hybrid polysaccharide–protein nature of JrPRP.

##### Microstructure Analysis

Scanning electron microscopy (SEM) was employed to investigate the surface morphology of JrPRP (Figure 3c). This technique enables high-resolution visualization of topographical features with enhanced depth of field, allowing detailed examination of the solid-state architecture of the protein-rich polysaccharide fraction. The SEM images acquired at 20 μm scale reveal a rough, porous, and sponge-like morphology characteristic of freeze-dried polysaccharide matrices (Figure 3c).

The presence of well-distributed pores and interconnected cavities is primarily attributed to the freeze-drying process, during which sublimation of ice crystals generates a three-dimensional porous network. Although this microstructure does not directly affect the biological activity assessed in solution-based assays, it provides valuable insight into the intrinsic macromolecular organization and intermolecular interactions within the polymer matrix.

Such porous architectures are widely reported for polysaccharide systems and are associated with high surface area, significant water retention capacity, and enhanced swelling behavior. These properties are particularly advantageous in food, cosmetic, and pharmaceutical applications, where hydration dynamics, structural stability, and solvent accessibility are critical parameters. The sponge-like morphology may facilitate solvent penetration and diffusion of active compounds, thereby potentially enhancing the functional performance of JrPRP in material-based formulations. Consistent with previous reports [32], the observed morphology supports its suitability for applications requiring high water absorption and efficient solvent interaction.

The coexistence of carbohydrate and protein fractions suggests the formation of a composite macromolecular assembly that may exhibit amphiphilic characteristics. Such systems are capable of forming supramolecular networks stabilized by hydrogen bonding and electrostatic interactions, which can influence solubility, molecular flexibility, and accessibility of reactive hydroxyl and carboxyl groups. The protein fraction may act as a structural modulator, affecting chain conformation and the exposure of functional groups involved in antioxidant and antimicrobial interactions.

Consequently, the functional behavior of JrPRP cannot be interpreted solely on the basis of monosaccharide composition but must be considered within the broader framework of its macromolecular organization. Taken together, compositional (HPLC), spectroscopic (UV–Vis and FT-IR), and morphological (SEM) analyses consistently demonstrate that JrPRP constitutes a uronic acid-rich, protein-associated acidic polysaccharide matrix organized into a hydrogen-bonded supramolecular network. This integrated structural configuration provides a coherent physicochemical basis for understanding the multifunctional biological activities described in subsequent sections.

### 3.3. Biological Activities of J. regia Root Bark Protein-Rich Polysaccharide

#### 3.3.1. Antioxidant Potential of Protein-Rich Polysaccharides from *J. regia* Root Bark

To elucidate the redox-modulating potential of JrPRP, multiple complementary in vitro assays were employed, including DPPH and ABTS radical scavenging, ferric reducing antioxidant power (FRAP), and ferrous ion (Fe^2+^) chelation (Table 2). The use of mechanistically distinct assays enables comprehensive evaluation of hydrogen atom transfer (HAT), single-electron transfer (SET), and metal-binding mechanisms.

##### Radical Scavenging Assays (DPPH, ABTS)

The DPPH assay evaluates the ability of antioxidants to donate hydrogen atoms or electrons to the stable DPPH radical, resulting in a decrease in absorbance after 30 min of incubation. JrPRP exhibited clear dose-dependent radical scavenging activity, with inhibition increasing from 11% to 70% across the tested concentration range (0.05–0.75 mg/mL). The calculated IC_50_ value was 405 ± 1.8 µg/mL (Table 2), indicating substantial hydrogen-donating capacity.

These results are consistent with previous reports on walnut-derived polysaccharides. Chemically modified *J. regia* shell polysaccharides, particularly acetylated and phosphorylated derivatives, have demonstrated enhanced antioxidant activity. Notably, phosphorylated fractions exhibited scavenging capacities of 92.3% against superoxide anions and 98.2% against hydroxyl radicals at 3.2 mg/mL, in some cases approaching or exceeding the activity of ascorbic acid [33]. This comparison suggests that structural modification strategies—especially the introduction of additional charged groups—can significantly amplify redox performance. Nevertheless, the present findings demonstrate that the native, non-derivatized JrPRP already possesses considerable intrinsic antioxidant capacity.

When compared with other plant-derived polysaccharides, JrPRP exhibits competitive and, in several cases, superior radical scavenging activity. For example, polysaccharides from almond gum (IC_50_ = 4 mg/mL), almond gum hemicellulose (5–6 mg/mL), guara fruit (10.8 mg/mL) [26,49], *Lupinus angustifolius* seeds (6.9 mg/mL) [49], and potato peels (2 mg/mL) [50] displayed weaker DPPH scavenging efficiency than JrPRP. It should be noted that synthetic antioxidants such as BHA often exhibit lower IC_50_ values (e.g., 0.5 mg/mL), reflecting their strong and rapid radical scavenging kinetics. However, rather than positioning JrPRP as superior to synthetic molecules, it is scientifically more appropriate to interpret it as a biologically compatible natural antioxidant system with moderate-to-strong radical scavenging efficiency and improved biocompatibility.

The ABTS radical cation decolorization assay further confirmed the antioxidant efficacy of JrPRP. This assay evaluates the ability of antioxidants to quench ABTS°^+^ radicals, indicated by a decrease in absorbance as the solution transitions from green-blue to colorless. JrPRP exhibited concentration-dependent scavenging activity, reaching 56% inhibition at 10 µg/mL, with an IC_50_ value of 225 ± 3.5 µg/mL (Table 2). The lower IC_50_ compared to DPPH suggests enhanced performance in aqueous-phase radical systems. This behavior likely reflects the highly hydrophilic and strongly anionic nature of JrPRP, which facilitates interaction with water-soluble radical species.

In comparison with other botanical sources, JrPRP demonstrated stronger ABTS scavenging capacity than polysaccharides from *Lupinus angustifolius* seeds (IC_50_ = 6.9 mg/mL) [49], potato peels (2 mg/mL) [50], and almond gum hemicellulose (5 mg/mL) [32]. However, JrPRP showed somewhat lower ABTS scavenging capacity than polysaccharides from *Rhamnus alaternus* leaves and stems, which achieved 79–80% inhibition at 10 µg/mL [6], indicating that antioxidant efficiency remains highly dependent on structural features such as monosaccharide composition, molecular weight, branching degree, and functional group density.

From a structural perspective, the antioxidant activity of JrPRP can be rationalized by the abundance of hydroxyl groups within the polysaccharide backbone, the high uronic acid content, providing dense carboxylate functionalities, and the presence of associated protein residues contributing electron-donating amino acid side chains. The predominance of uronic acid likely enhances redox behavior through increased solubility, improved radical accessibility, and potential metal-binding stabilization. Moreover, the composite polysaccharide–protein architecture may promote synergistic effects by improving conformational flexibility and exposing reactive functional groups within the macromolecular network.

In line with these structural features, previous studies have demonstrated that a high content of uronic acid has been strongly associated with enhanced antioxidant activity, possibly due to the susceptibility of uronic acid chains to free radical-induced cleavage. Furthermore, monosaccharides such as mannose and rhamnose have been individually linked to tumor-inhibitory and antioxidant activities. However, despite these correlations, the precise structure–activity relationships and underlying mechanisms remain insufficiently elucidated and warrant further investigation [15].

Overall, JrPRP demonstrates competitive antioxidant performance within the range reported for natural acidic polysaccharides. Its activity arises from cooperative interactions between carbohydrate and protein domains rather than from a single structural determinant, reinforcing the structure–function coherence established in the physicochemical characterization section.

##### Determination of Antioxidant Activity Using the Ferric Reducing Antioxidant Power (Frap) Assay

The electron-donating ability of JrPRP was further evaluated using the FRAP assay. JrPRP exhibited a strong reducing capacity, with IC_50_ of 338.3 ± 2.1 µg/mL (Table 2), confirming its ability to reduce Fe^3+^ to Fe^2+^ via a single-electron transfer mechanism. This property is critical in preventing oxidative damage and supports the potential application of JrPRP in therapies targeting oxidative stress-related disorders.

Phosphorylated polysaccharides derived from *J. regia* shells demonstrated FRAP values comparable to those of ascorbic acid at high concentrations [33], reinforcing the idea that chemical derivatization can significantly enhance the antioxidant power of natural polysaccharides, including JrPRP.

When compared to other plant sources, JrPRP showed superior reducing capacity. Polysaccharides from *Rhamnus alaternus* exhibited significantly weaker activity, with IC_50_ = 141.7 mg/mL for stems and IC_50_ = 203.8 mg/mL for leaves [6]. Other natural sources, such as *Schinus terebinthifolius* and *S. molle* fruits, showed even lower reducing powers, with values of 0.3 and 0.2 at 400 µg/mL, respectively [37]. Polysaccharides extracted from pine seeds using acetone showed higher reducing ability (IC_50_ = 46.4 ± 5.2 µg/mL), while ethanol and propanol extracts were less effective (IC_50_ = 164.5 ± 18.1 µg/mL and IC_50_ = 173.7 ± 0.4 µg/mL, respectively) [27]. In contrast, pistachio shell polysaccharides showed minimal reducing effects (0.04–0.2 at 400 µg/mL) [9].

The reducing behavior can be attributed to several structural features, including abundant hydroxyl groups within pyranose rings, a high density of carboxylate groups originating from uronic acid residues, and associated protein domains that may provide electron-rich amino acid side chains.

Reducing capacity in polysaccharides is influenced by chain flexibility, branching degree, and accessibility of reactive sites. In JrPRP, the coexistence of acidic groups and protein-associated domains may facilitate electron transfer through hydrogen-bonded networks and transient coordination with ferric ions.

When compared with other plant-derived polysaccharides, the FRAP activity of JrPRP falls within the upper range of naturally occurring acidic polysaccharides. The variations in reducing power reported across species can be largely attributed to differences in molecular weight, monosaccharide composition, degree of branching, and physicochemical properties, including solubility and viscosity, which are recognized as key determinants of antioxidant activity [51].

##### Ferrous (Fe^2+^) Ion Chelating Activity

Metal ion chelation represents a distinct antioxidant mechanism, as transition metals catalyze Fenton-type reactions that generate highly reactive hydroxyl radicals and play a crucial role in the onset of oxidative stress. JrPRP demonstrated pronounced Fe^2+^ chelating capacity, with an IC_50_ value of 229.8 ± 1.7 µg/mL (Table 2). This result suggests a strong affinity for metal ion binding, potentially inhibiting metal-catalyzed free radical formation. This chelating behavior can be attributed to the high density of carboxylate groups derived from uronic acid residues, the involvement of hydroxyl functionalities in coordination, and the possible contribution of protein-associated amino and carbonyl groups. This property not only limits metal-catalyzed radical generation but also reinforces the functional relevance of JrPRP in biological systems where oxidative stress is mediated by transition metals.

Comparable chelation capacities have been reported for polysaccharides from *Rhamnus alaternus* showed similar chelation capacities, with IC_50_ = 219 µg/mL and 224 µg/mL for stems and leaves, respectively [6], whereas other natural sources such as *L. sordida* mycelia (5.1 mg/mL), pistachio shells (IC_50_ = 3.4 mg/mL), and almond gum (IC_50_ = 0.2 mg/mL) [23,32,52] exhibited weaker activity. Phosphorylated *J. regia* shell polysaccharides also exhibited high Fe^2+^ chelation at 3.2 mg/mL, performing comparably to vitamin C [33], suggesting that phosphate group incorporation enhances metal-binding affinity and antioxidant effectiveness.

When considered collectively, the antioxidant assays demonstrate that JrPRP operates through multiple complementary mechanisms: Hydrogen atom donation (DPPH), Radical cation quenching in aqueous systems (ABTS), Single-electron transfer reducing activity (FRAP) and Transition metal sequestration (Fe^2+^ chelation).

This multifunctionality is strongly supported by the macromolecular architecture characterized earlier, a uronic acid-rich backbone providing dense carboxylate groups and strong anionic character, a protein association (~34% *w*/*w*), potentially enhancing conformational flexibility and redox synergy, and a hydrogen-bonded supramolecular network stabilizing reactive intermediates.

Therefore, the antioxidant behavior of JrPRP emerges from the cooperative interplay between carbohydrate and protein domains within an acidic macromolecular framework, rather than from a single structural determinant.

Importantly, JrPRP should not be positioned as superior to synthetic antioxidants; rather, it represents a biologically compatible natural redox system with moderate-to-strong radical scavenging and metal-binding efficiency. This integrated redox profile supports its potential application in nutraceutical and biomedical contexts targeting oxidative stress. The chelation capacity observed here aligns with the structural identity of JrPRP as an acidic polysaccharide matrix and reinforces the mechanistic coherence between composition and biological function.

#### 3.3.2. Antibacterial Activities

##### Minimum Inhibitory Concentration (MIC) and Minimum Bactericidal Concentration (MBC)

The antibacterial potential of JrPRP was evaluated using the broth microdilution method to determine both its inhibitory (MIC) and bactericidal (MBC) effects against a panel of pathogenic bacterial strains. The MIC corresponds to the lowest concentration of the extract capable of inhibiting visible bacterial growth after 18 h of incubation at 37 °C, whereas the MBC represents the lowest concentration able to kill the bacteria. Together, these parameters provide important insights into whether the antimicrobial effect is bacteriostatic or bactericidal.

##### Antibacterial Efficacy Across Strains

JrPRP was tested against six reference bacterial strains, and the results are summarized in Table 3. The MIC values ranged from 2 to 9 mg/mL, indicating variable sensitivity among the tested microorganisms. Among them, *Pseudomonas aeruginosa* was the most sensitive strain, with MIC and MBC values of 2 and 3 mg/mL, respectively, whereas *Bacillus cereus* exhibited the highest resistance (MIC = 9 mg/mL, MBC = 10 mg/mL).

Based on MIC values, the order of bacterial susceptibility to JrPRP was:

*P. aeruginosa* < *E. coli*, *Enterobacter* sp. < *S. aureus* < *K. pneumoniae* < *B. cereus*.

The close correspondence between MIC and MBC values indicates that the extract does not merely inhibit bacterial growth but is capable of exerting a bactericidal effect. Such activity is commonly observed for natural polysaccharides, which often exhibit moderate antibacterial potency but high biocompatibility and safety.

##### Mbc/mic Ratios: Confirmation of Bactericidal Activity

The MBC/MIC ratio is commonly used to distinguish bactericidal (≤2) from bacteriostatic (>2) effects. As shown in Table 3, all tested strains exhibited ratios below 2, confirming the bactericidal activity of JrPRP.

The lowest ratios were observed for *B. cereus* and *K. pneumoniae* (1.1), indicating particularly strong bactericidal effects. Slightly higher but still bactericidal ratios (1.4–1.5) were recorded for *S. aureus*, *E. coli*, *Enterobacter* sp., and *P. aeruginosa*. The consistent MBC/MIC ratios below the bactericidal threshold further highlight the antimicrobial potential of JrPRP.

##### Structural Features and Possible Mechanisms of Antibacterial Activity

The antibacterial activity of plant-derived polysaccharides is generally attributed to multiple complementary mechanisms, including disruption of bacterial cell walls and membranes, interference with key metabolic pathways, inhibition of nutrient transport, and impairment of biofilm formation. These actions ultimately compromise bacterial viability and proliferation. In general, Gram-positive bacteria tend to be more susceptible to antimicrobial agents because they lack the outer membrane that characterizes Gram-negative species, allowing easier access to their peptidoglycan-rich cell walls. Conversely, Gram-negative bacteria are typically more resistant due to the presence of an additional outer membrane that restricts the diffusion of antimicrobial molecules, particularly large or hydrophobic compounds [53,54,55].

Interestingly, our findings revealed a notable exception to this general trend: *Pseudomonas aeruginosa*, a Gram-negative bacterium, exhibited the highest sensitivity to JrPRP. This observation suggests that the antimicrobial activity of JrPRP may involve mechanisms beyond simple membrane permeability disruption. In contrast, *Bacillus cereus* (Gram-positive) showed the highest resistance, indicating that bacterial susceptibility is influenced not only by Gram classification but also by other factors such as membrane composition, efflux pump activity, metabolic adaptability, and biofilm-forming capacity.

The antibacterial efficacy of polysaccharides is also strongly influenced by their structural characteristics, including molecular weight, degree of branching, and monosaccharide composition, which govern their interaction with bacterial surfaces and membrane components [54].

In the present study, several structural features of JrPRP may contribute to its antimicrobial behavior. First, the extract is characterized by a high uronic acid content, which confers a strong polyanionic character to the polysaccharide backbone. This high density of negatively charged groups may promote electrostatic interactions with positively charged bacterial surface proteins and membrane-associated components, potentially destabilizing membrane-associated processes or interfering with ion transport mechanisms.

In addition, JrPRP exhibited notable metal-chelating capacity, which may further contribute to its antimicrobial activity by sequestering essential metal ions required for bacterial enzymatic reactions and metabolic functions. Deprivation of these cofactors can disrupt cellular homeostasis and indirectly inhibit microbial growth.

The acidic and hydrophilic nature of JrPRP may also facilitate its binding to bacterial cell envelopes, potentially interfering with membrane-associated proteins or ion homeostasis. Unlike small hydrophobic antimicrobial molecules that directly disrupt lipid bilayers, polysaccharide-based systems generally exert their activity through surface interactions, electrostatic binding, and metabolic perturbation rather than direct membrane lysis. This mode of action is consistent with the behavior of many natural polysaccharides reported in the literature.

Furthermore, spectroscopic analyses revealed the presence of a protein–polysaccharide architecture, which may impart amphiphilic properties to the macromolecule and enhance its interaction with bacterial cell surfaces. Such hybrid macromolecular systems may facilitate binding to membrane proteins or surface receptors, thereby affecting cellular processes essential for bacterial survival.

Notably, the relatively moderate MIC values observed in this study (mg/mL range) are consistent with those commonly reported for natural polysaccharides, which typically exhibit moderate intrinsic antibacterial activity but high levels of biocompatibility. Despite this moderate potency, their ability to act through multiple mechanisms and their low toxicity make them attractive candidates for biomedical and biotechnological applications.

Similar antibacterial behaviors have been reported for other plant- and marine-derived polysaccharides. For example, polysaccharides isolated from *Phormidium versicolor* and *Alsidium corallinum* have demonstrated inhibitory effects against both Gram-positive and Gram-negative bacteria, including *Escherichia coli*, *Staphylococcus aureus*, and *Pseudomonas aeruginosa* [53,56,57], supporting the relevance of polysaccharide-based antimicrobial systems.

Overall, the results of this study demonstrate that JrPRP exhibits broad-spectrum bactericidal activity and suggest that its antimicrobial properties are closely associated with its distinctive structural features, particularly its uronic acid-rich backbone and protein-associated macromolecular organization. These characteristics are likely to contribute to a multifactorial antibacterial mechanism involving electrostatic interactions with microbial surfaces, sequestration of metal ions, and interference with membrane-associated processes, ultimately leading to metabolic disruption and inhibition of microbial proliferation.

#### 3.3.3. Antibiofilm Activity

Biofilm formation represents a major virulence strategy used by pathogenic bacteria to survive hostile environments, evade host immune responses, and develop resistance to antimicrobial agents. Consequently, the inhibition of biofilm formation has become an important objective in the development of alternative antimicrobial strategies. Plant-derived polysaccharides have attracted increasing attention due to their ability to interfere with different stages of biofilm development, including bacterial adhesion, quorum sensing, extracellular polymeric substance (EPS) production, and cell–cell communication [6].

**Figure 4:**
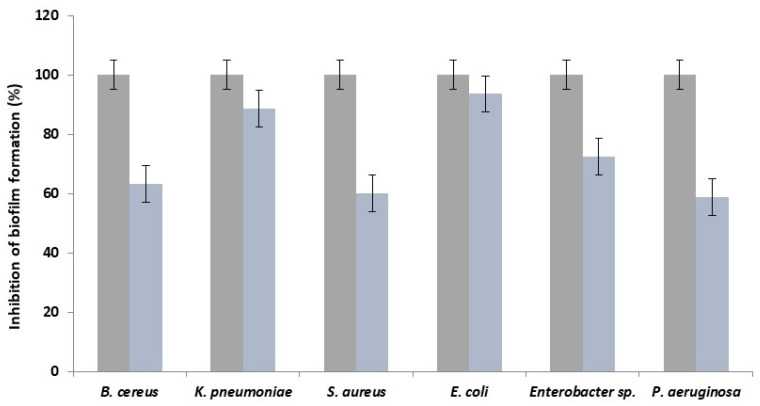
Effect of JrPRP (■) on the binding of pathogenic bacteria expressed as a percentage of inhibition evaluated by the crystal violet test (positive control (■)).

In the present study, protein-rich polysaccharides extracted from the root bark of *J. regia* (JrPRP) were evaluated for their ability to inhibit biofilm formation in several pathogenic bacterial strains. The results revealed pronounced antibiofilm activity, with inhibition rates ranging from 58% to 94% depending on the strain and concentration (Figure 4). The strongest inhibition was observed for *E. coli* and *K. pneumoniae*, with biofilm inhibition rates of 94% and 88%, respectively, at concentrations of 4 and 6 mg/mL. For *P. aeruginosa*, *S. aureus*, *B. cereus*, and *Enterobacter* sp., inhibition rates of 58%, 59%, 63%, and 72% were recorded at concentrations of 2, 5, 9, and 4 mg/mL, respectively. Although *P. aeruginosa* exhibited comparatively lower susceptibility, the overall results demonstrate that JrPRP is capable of significantly reducing biofilm formation across both Gram-positive and Gram-negative bacterial species.

The antibiofilm activity of JrPRP may arise from several complementary mechanisms. Polysaccharides can interfere with the early stages of biofilm development by preventing bacterial adhesion to surfaces, disrupting extracellular polymeric substances, or competing with carbohydrate-binding proteins involved in cell attachment. In particular, the presence of uronic acids and neutral sugars may hinder the initial attachment phase of biofilm formation, which represents a critical step in biofilm establishment. By disrupting early adhesion events, JrPRP may effectively prevent the maturation and stabilization of bacterial biofilms.

The remarkable antibiofilm activity observed in this study may also be closely related to the distinctive structural composition of JrPRP, particularly its high uronic acid content. This elevated density of negatively charged carboxyl groups confers a strong polyanionic character to the polysaccharide backbone, which may promote electrostatic interactions with positively charged bacterial surface proteins and lectins involved in adhesion processes. Such interactions may interfere with carbohydrate–lectin recognition events that are essential for bacterial attachment and biofilm stabilization.

Furthermore, JrPRP demonstrated notable metal-chelating capacity, which may further contribute to antibiofilm activity by sequestering essential metal ions required for bacterial enzymatic processes, quorum sensing pathways, and oxidative stress regulation. The deprivation of these cofactors can disrupt metabolic homeostasis and limit the ability of bacteria to establish stable biofilm structures.

The superior antibiofilm performance of JrPRP compared with previously reported plant polysaccharides further supports its potential as an effective biofilm-inhibiting agent. For example, polysaccharides isolated from *Rhamnus alaternus* leaves (WSPRaL) and stems (WSPRaS) showed inhibition rates of approximately 70% and 81%, respectively, against *K. pneumoniae*, while other strains exhibited considerably lower susceptibility [6]. In contrast, JrPRP achieved inhibition levels approaching 94%, suggesting stronger or more specific interactions with bacterial biofilm-associated structures.

In addition, the protein–polysaccharide architecture identified by spectroscopic analyses may enhance the interaction of JrPRP with bacterial surfaces through amphiphilic properties, facilitating binding to membrane proteins or extracellular matrix components involved in biofilm stabilization. Such hybrid macromolecular systems may therefore interfere with both bacterial adhesion and matrix cohesion.

Importantly, while many plant-derived polysaccharides display moderate antibacterial effects, strong antibiofilm activity is less frequently reported. The combination of bactericidal activity and pronounced biofilm inhibition observed in this study highlights the functional relevance of JrPRP as a multifunctional antimicrobial biopolymer.

These observations highlight a clear structure–activity relationship, in which the high uronic acid content and the protein–polysaccharide architecture of JrPRP appear to play a central role in modulating its antibiofilm properties.

Taken together, these findings suggest that JrPRP inhibits biofilm formation through a multifactorial mechanism involving electrostatic surface interactions, metal ion sequestration, and disruption of carbohydrate-mediated adhesion processes. Such properties make this uronic acid-rich protein–polysaccharide complex a promising candidate for applications aimed at preventing biofilm-associated infections in biomedical, pharmaceutical, and food-preservation systems [54].

Overall, the combined antibacterial and antibiofilm activities observed for JrPRP emphasize its potential as a multifunctional antimicrobial biopolymer. While its antibacterial activity contributes to the inhibition of planktonic bacterial growth, the pronounced antibiofilm effect indicates an additional capacity to interfere with bacterial adhesion and biofilm development. This dual mode of action is particularly valuable, as biofilm-associated microorganisms exhibit increased tolerance to conventional antimicrobial agents [54]. Therefore, the ability of JrPRP to target both bacterial proliferation and biofilm formation may significantly enhance its applicability in biomedical, pharmaceutical, and food-preservation systems.

#### 3.3.4. Antihemolytic and Anticoagulant Activities

##### Hemocompatibility Assessment of JrPRP

Hemolytic Activity on Blood Agar

The hemolytic potential of protein-rich polysaccharides extracted from *J. regia* root bark (JrPRP) was evaluated using blood agar plates at concentrations ranging from 30 to 120 mg/mL. After 24 h of incubation, no visible hemolytic halos were observed, indicating the absence of erythrocyte membrane disruption and confirming the good hemocompatibility of JrPRP. These observations are consistent with previous reports describing negligible hemolytic activity for polysaccharides isolated from *Coriolus versicolor* and *Panax ginseng* [58,59], supporting the favorable safety profile of plant-derived macromolecules. In contrast, certain marine sulfated polysaccharides, such as those extracted from *Gracilaria verrucosa*, may exhibit variable hemolytic effects depending on sulfate content, molecular weight, and extraction conditions [60].

The absence of hemolytic activity, even at relatively high concentrations, indicates that JrPRP exhibits minimal interaction with erythrocyte membranes. This behavior may be attributed to its non-sulfated structure and the predominance of uronic acid residues, which impart a moderate polyanionic character without inducing the pronounced membrane disruption commonly associated with highly sulfated polysaccharides. These properties are particularly advantageous for biomedical applications involving direct blood contact.

Cytoprotective Effects on Erythrocytes

**Figure 5:**
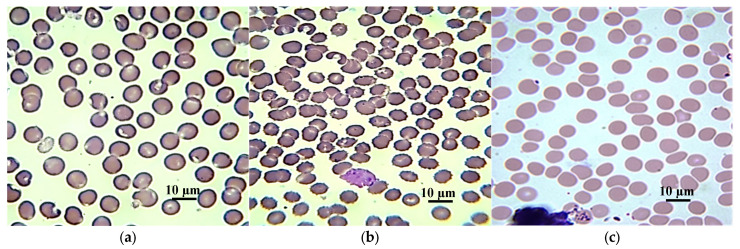
Blood smear of erythrocytes observed under magnification (G × 1000): (**a**) Control erythrocytes displaying a normal discoid morphology, (**b**) Erythrocytes exposed to H_2_O_2_ showing transformation into echinocytes with membrane deformations, and (**c**) Erythrocytes co-treated with protein-rich polysaccharides from *J. regia* root bark (JrPRP), demonstrating preservation of cellular structure.

The cytoprotective effects of JrPRP against oxidative damage were evaluated through morphological analysis of erythrocytes using May–Grünwald–Giemsa-stained blood smears. As shown in Figure 5a, untreated erythrocytes displayed their characteristic biconcave discoid morphology with smooth membranes, indicating normal cellular integrity.

Exposure to hydrogen peroxide (H_2_O_2_), a well-known inducer of oxidative stress, caused pronounced morphological alterations characterized by the formation of echinocytes (Figure 5b). These cells exhibit spiculated and crenated membranes, reflecting oxidative damage to erythrocyte membranes.

Remarkably, co-treatment with JrPRP (300 mg/mL) largely preserved erythrocyte morphology (Figure 5c), with most cells maintaining their normal discoid structure. This observation demonstrates a protective effect against H_2_O_2_-induced oxidative membrane injury.

The cytoprotective activity of JrPRP may be attributed to several complementary mechanisms, including stabilization of erythrocyte lipid bilayers, scavenging of reactive oxygen species, electrostatic interactions between uronic acid residues and membrane components, and potential shielding effects associated with the protein–polysaccharide architecture. Echinocyte formation is widely recognized as a marker of oxidative cytotoxicity [26]. Therefore, the preservation of erythrocyte morphology strongly supports both the antioxidant potential and hemocompatibility of JrPRP.

Antihemolytic Activity Against NaCl-Induced Hemolysis

**Figure 6:**
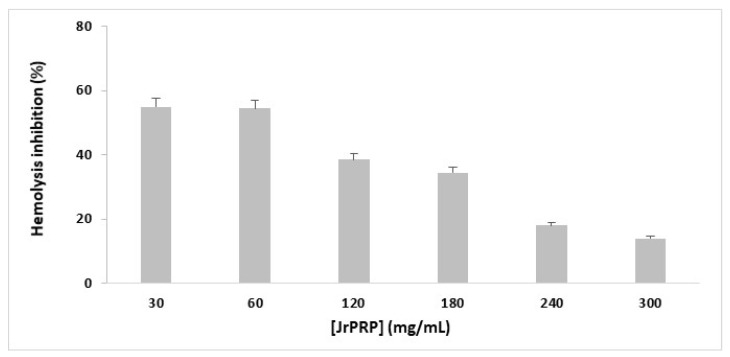
Percentage of hemolysis inhibition of JrPRP.

The protective effect of JrPRP against erythrocyte lysis was further investigated using hypotonic NaCl-induced hemolysis [27]. JrPRP exhibited significant antihemolytic activity, with inhibition rates ranging from 27% to 58% at concentrations between 30 and 300 mg/mL (Figure 6).

Importantly, total hemolysis remained below 10% across all tested concentrations, confirming the low intrinsic toxicity of the polysaccharide extract. The preservation of intact discoid erythrocytes without echinocyte formation further supports the favorable hemocompatibility profile of JrPRP.

Comparable protective effects have been reported for other plant-derived polysaccharides. For instance, polysaccharides from *Annona muricata* leaves inhibited erythrocyte lysis under hypotonic conditions with protection rates reaching approximately 60%, depending on molecular weight fractions [55]. Similarly, extracts from *Pinus halepensis* seeds demonstrated significant antihemolytic activity, highlighting the capacity of plant polysaccharides to stabilize erythrocyte membranes under stress conditions [61,62].

Variations in the antihemolytic efficacy of polysaccharides are generally associated with structural parameters such as molecular weight distribution, charge density, and monosaccharide composition, which govern their interactions with membrane lipids and ion transport processes [27]. In the case of JrPRP, the elevated uronic acid content may promote electrostatic stabilization of erythrocyte membranes, thereby reducing osmotic fragility and maintaining membrane integrity.

Collectively, these findings confirm that JrPRP exhibits low hemolytic activity together with significant membrane-stabilizing capacity, indicating a favorable hemocompatibility profile. These hemocompatibility results provide an essential prerequisite for evaluating the anticoagulant potential of JrPRP, as safe interactions with erythrocytes are a key requirement for blood-contacting biomolecules.

##### Anticoagulant Activity of JrPRP

The anticoagulant activity of the protein-rich polysaccharide extracted from *J. regia* root bark (JrPRP) was evaluated using two complementary in vitro assays: prothrombin time (PT) and activated partial thromboplastin time (aPTT). The PT assay reflects the extrinsic coagulation pathway, primarily involving factors VII, X, V, prothrombin, and fibrinogen, whereas the aPTT assay evaluates the intrinsic pathway, including factors XII, XI, IX, VIII, X, V, prothrombin, and fibrinogen [63]. These assays are widely used to assess the ability of natural polysaccharides to interfere with the coagulation cascade.

JrPRP induced a clear dose-dependent prolongation of prothrombin time (PT) compared to the control (12.5 s). PT values increased progressively with concentration, reaching 19.1 s at 2.5 µg/mL (Table 4), indicating a marked effect on the extrinsic coagulation pathway. This trend was further confirmed by the PT ratio, which increased from 1.00 to 1.53. Importantly, the calculated international normalized ratio (INR, ISI = 1.3) also showed a consistent increase from 1.00 in the control to approximately 1.70 at the highest concentration, providing a standardized confirmation of the anticoagulant effect.

Although sulfated polysaccharides from marine organisms such as *Sciaena umbra* or plant sources including *Rhamnus alaternus* and *Pinus halepensis* have been reported to induce stronger prolongation of coagulation times [6,27,53], JrPRP exhibited a moderate yet significant activity. Based on FTIR analysis, no definitive evidence of sulfation was established; therefore, the observed anticoagulant effect is more likely governed by alternative structural features.

The anticoagulant behavior of JrPRP can be primarily attributed to its high uronic acid content, which imparts a pronounced polyanionic character to the polysaccharide matrix. Uronic acid-rich polysaccharides are known to interact electrostatically with positively charged domains of coagulation factors, thereby interfering with their activation and assembly within the coagulation cascade. In this context, the high density of carboxylate groups (–COO^−^) in JrPRP likely enhances these interactions, contributing to the observed delay in clot formation. Although JrPRP is not chemically sulfated, its charge-driven behavior may be functionally comparable to that of highly anionic sulfated polysaccharides such as fucans and heparin, whose anticoagulant activity is strongly associated with high negative charge density [64]. These findings highlight the central role of carboxylate-mediated electrostatic interactions in governing the anticoagulant potential of JrPRP.

The influence of JrPRP on the intrinsic coagulation pathway was further examined using the aPTT assay. JrPRP induced a marked prolongation of aPTT values even at a minimal concentration of 0.5 µg/µL, confirming its inhibitory effect on intrinsic pathway components. Similar anticoagulant behaviors have been reported for uronic acid-containing or protein-associated polysaccharides. For example, polysaccharides isolated from *Marsypianthes chamaedrys* significantly prolonged aPTT values, while plant-derived polysaccharides from *Apocynum venetum* flowers also exhibited measurable anticoagulant effects [53,63,65].

The anticoagulant properties of natural polysaccharides are known to depend on structural parameters such as molecular weight, monosaccharide composition, charge density, and conformational flexibility [27]. In the case of JrPRP, the predominance of uronic acid residues, combined with the presence of protein-associated domains, may enhance molecular recognition and binding affinity toward plasma proteins and coagulation factors. Furthermore, the protein–polysaccharide architecture may promote additional interactions with plasma components, thereby strengthening its capacity to modulate coagulation processes [27].

Overall, JrPRP exhibits a moderate yet significant anticoagulant effect, with a more pronounced influence on the intrinsic pathway while maintaining limited interference with the extrinsic pathway. This balanced activity may be advantageous for biomedical applications, as it may reduce the risk of excessive bleeding commonly associated with highly potent anticoagulant agents. In addition, the previously demonstrated hemocompatibility and low hemolytic activity of JrPRP further support its favorable safety profile. Taken together, these findings suggest that protein-rich polysaccharides derived from *J. regia* root bark represent promising natural biomolecules for the development of blood-compatible biomaterials and anticoagulant therapeutic formulations.

##### Inhibitory Effects on Human Platelet Aggregation

**Figure 7:**
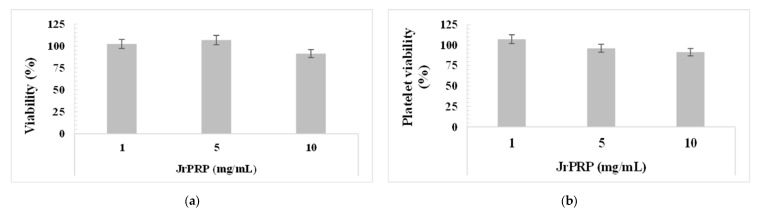
(**a**) Platelet viability in the presence of different JrPRP concentrations and (**b**) Cytotoxic effect of JrPRP tested at different concentrations on the HEK-293 cell line. The results are presented as a percentage of cell survival.

To evaluate its hematological safety, the effects of *J. regia* root bark protein-rich polysaccharides (JrPRP) on human platelets were investigated. Platelet viability remained at 100% at a concentration of 1 mg/mL, while at higher concentrations of 5 and 10 mg/mL, viability was maintained at approximately 90% (Figure 7a), indicating the absence of cytotoxic effects. These results demonstrate that JrPRP does not compromise platelet integrity or metabolic activity even at relatively high concentrations.

Comparable findings have been reported for plant-derived polysaccharides from *Ganoderma lucidum* and *Astragalus membranaceus*, which preserved platelet viability while exhibiting immunomodulatory and anti-inflammatory activities without inducing hemolysis or thrombosis [33,66]. In contrast, certain sulfated marine polysaccharides such as fucoidans have been reported to induce dose-dependent platelet aggregation, highlighting the critical influence of molecular structure and charge density on hemocompatibility [64,67].

The absence of platelet cytotoxicity observed for JrPRP may be attributed to its non-sulfated structure and the predominance of carboxyl groups rather than sulfate esters, which likely results in milder electrostatic interactions with platelet membranes. In addition, the protein–polysaccharide architecture may provide steric stabilization that limits direct membrane disruption. Collectively, these findings support the favorable hemocompatibility profile of JrPRP and reinforce its potential for systemic biomedical applications where hematological safety is essential.

##### Cytotoxicity Tests on Hek-293 Non-Cancerous Cells

The cytotoxicity of JrPRP toward non-cancerous human cells was evaluated using the MTT assay on HEK-293 cells. After 24 h of exposure, cell viability exceeded 100% at concentrations up to 1 mg/mL, while approximately 90% viability was maintained at 10 mg/mL (Figure 7b). These results confirm the low cytotoxicity of JrPRP toward normal cells and are consistent with previous studies on plant-derived protein-rich polysaccharides [68,69].

Interestingly, several studies have shown that structurally related polysaccharides may exhibit selective biological activity, demonstrating low toxicity toward normal cells while inhibiting the proliferation of cancer cell lines. For example, polysaccharides extracted from barley grass inhibited the growth of HT29, Caco-2, and CT26.WT cancer cells while maintaining high viability in normal cells [69]. Similarly, protein-rich polysaccharides derived from *J. regia* bark were reported to suppress the proliferation of LoVo colon cancer cells while preserving the viability of normal NCM460 cells [70].

Taken together, these observations suggest that JrPRP possesses a favorable safety profile combined with potential selective bioactivity. This dual characteristic highlights its relevance as a natural bioactive polymer with promising applications in functional foods, nutraceutical formulations, and biomedical therapies.

#### 3.3.5. In Vitro Anti-Inflammatory Properties

##### Egg Albumin Denaturation Assay

**Figure 8:**
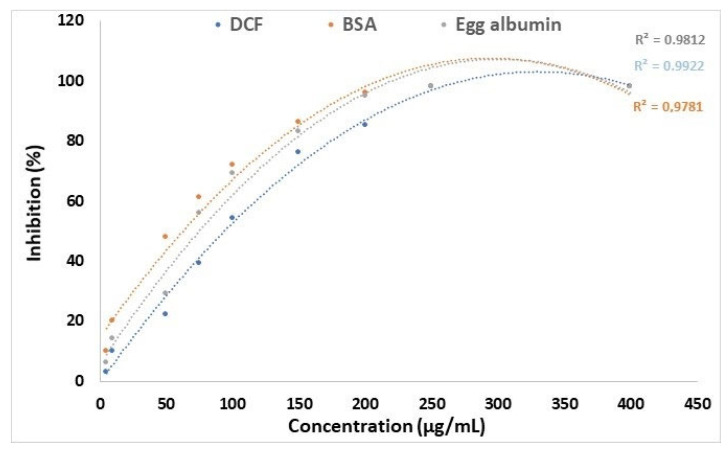
Concentration-dependent inhibition of protein denaturation by the protein-rich polysaccharide extract from *J. regia* root bark (JrPRP) evaluated using egg albumin (--●--) and bovine serum albumin (--●--) assays. JrPRP exhibited strong anti-inflammatory activity, reaching 98% inhibition at 200 µg/mL, comparable to the reference drug Diclofenac (--●--). Data are expressed as mean ± SD (*n* = 3). Statistical significance was determined relative to the control (*p* < 0.05).

The protein-rich polysaccharide extract obtained from the root bark of *J. regia* (JrPRP) exhibited pronounced anti-inflammatory activity by effectively inhibiting egg albumin denaturation. At a concentration of 200 µg/mL, JrPRP achieved 98% inhibition, indicating a strong capacity to stabilize protein structures under stress conditions (Figure 8).

The inhibition profile showed a clear dose-dependent trend, with increasing concentrations resulting in progressively higher inhibition until reaching a plateau between 250 and 300 µg/mL, followed by a slight decrease. This behavior suggests a saturation phenomenon commonly observed in macromolecular interactions between polysaccharides and proteins, where binding sites become progressively occupied.

The egg albumin denaturation assay is widely recognized as a reliable in vitro model for evaluating anti-inflammatory activity, as protein denaturation represents a key biochemical event associated with inflammatory processes. During inflammation, proteins undergo conformational destabilization, which contributes to cellular damage and immune activation. Consequently, compounds capable of preventing or reducing protein denaturation may help mitigate inflammation-related damage. The strong inhibition observed for JrPRP therefore indicates a significant protein-stabilizing capacity that may underlie its anti-inflammatory potential. Comparable activities have been reported for polysaccharides from *Moringa oleifera*, which have also demonstrated protective effects against albumin denaturation [71].

Furthermore, the inhibition profile of JrPRP was comparable to that of the reference anti-inflammatory drug diclofenac, reinforcing the effectiveness of the polysaccharide. The high R^2^ values (0.98 for egg albumin and 0.99 for diclofenac) confirm the strong correlation between concentration and inhibition, highlighting the consistency and reproducibility of JrPRP in preventing protein denaturation.

##### Bovine Serum Albumin Denaturation Assay

The anti-inflammatory activity of JrPRP was further confirmed using the bovine serum albumin (BSA) denaturation assay, which serves as a complementary model for assessing the protective effects of bioactive compounds on protein stability. Protein denaturation is a critical event during inflammatory responses, as structural alterations can lead to tissue damage and amplification of immune reactions. The ability of JrPRP to stabilize protein structures therefore supports its potential role in modulating inflammation through preservation of secondary and tertiary conformations.

At 200 µg/mL, JrPRP achieved 98% inhibition of BSA denaturation, slightly exceeding the inhibition observed for diclofenac (96%) under identical experimental conditions (Figure 8). Similar to the egg albumin assay, the inhibition curve displayed a clear concentration-dependent increase, reaching maximal activity between 200 and 250 µg/mL, followed by a slight decrease at higher concentrations. The high R^2^ values (>0.97) further confirm the strong dose–response relationship and the reliability of the experimental data.

The anti-denaturation activity of JrPRP may be explained by multiple molecular interactions, including hydrogen bonding with amino acid residues such as threonine, lysine, and tyrosine, as well as electrostatic interactions mediated by uronic acid groups present in the polysaccharide structure. In addition, the protein–polysaccharide architecture of JrPRP may provide steric stabilization, reducing conformational unfolding of proteins under stress conditions. Similar mechanisms have been documented for plant-derived polysaccharides, which are well known for their broad spectrum of biological activities, including anti-inflammatory, antitumor, and immunomodulatory effects [64].

##### Integrated Interpretation of Anti-Inflammatory Activity

Overall, the results obtained from both albumin denaturation assays consistently demonstrate that JrPRP possesses strong anti-inflammatory potential. This activity is closely associated with its compositional and structural characteristics, particularly its high uronic acid content and the presence of protein-associated domains, which together confer a balanced polyanionic character and a well-defined macromolecular architecture. These features enhance the ability of JrPRP to interact with proteins through hydrogen bonding, electrostatic interactions, and steric stabilization, thereby limiting conformational destabilization under stress conditions.

From a macromolecular perspective, JrPRP can be regarded as a naturally occurring functional biopolymer combining carbohydrate and protein moieties. Its physicochemical properties underpin not only its anti-inflammatory activity but also its previously demonstrated antioxidant, antibacterial, antibiofilm, hemocompatible, and anticoagulant effects. Such multifunctionality is commonly observed in uronic acid-rich polysaccharides and is attributed to their capacity to engage in diverse molecular interactions with reactive oxygen species, plasma proteins, cellular membranes, and microbial surfaces.

The coexistence of these biological activities suggests a synergistic biofunctional profile driven by the polyanionic nature and structural complexity of JrPRP. This enables simultaneous protein stabilization, modulation of oxidative processes, metal ion chelation, and interference with microbial adhesion and biofilm formation. In addition, its low hemolytic activity and good hemocompatibility further support its favorable safety profile for potential biomedical applications.

Taken together, these findings highlight JrPRP as a multifunctional natural biopolymer with broad-spectrum biological activities. Its unique protein–polysaccharide architecture, high uronic acid content, and associated functional properties position it as a promising candidate for the development of advanced biomaterials and functional formulations in biomedical, pharmaceutical, nutraceutical, and food-preservation systems, where both bioactivity and biocompatibility are essential.

## 4. Conclusions

This study presents the first comprehensive structural and functional characterization of protein-rich polysaccharides isolated from the root bark of *Juglans regia* (JrPRP), identifying them as a promising class of naturally derived multifunctional biopolymers.

The compositional profile of JrPRP, characterized by the presence of uronic acid-related components and protein-associated domains, suggests that these structural features may contribute to its observed biological properties, supporting a potential structure–function relationship.

Functionally, JrPRP exhibited anticoagulant activity affecting both intrinsic and extrinsic coagulation pathways while maintaining favorable hemocompatibility. The low hemolytic activity, preservation of erythrocyte morphology under oxidative stress, and absence of platelet cytotoxicity collectively support its biocompatibility. Although JrPRP does not appear to contain sulfate groups, its anticoagulant effect may be associated with carboxyl groups derived from uronic acid residues, as well as its polysaccharide–protein architecture, which may facilitate electrostatic interactions with coagulation factors.

In addition, JrPRP demonstrated antibacterial and antibiofilm activities, particularly against *Staphylococcus aureus*. These effects may involve multiple mechanisms, including interference with bacterial adhesion and disruption of biofilm development. The lower activity observed against Gram-negative bacteria such as *Pseudomonas aeruginosa* may be attributed to the protective role of the outer membrane. Furthermore, the observed antihemolytic and cytoprotective effects reinforce the overall biocompatibility of this fraction.

Overall, this study provides new insights into the compositional characteristics and biological potential of protein-rich polysaccharides derived from J. regia root bark. JrPRP may be considered a promising natural biopolymer in which charge properties and macromolecular organization contribute to biological activity.

However, it is important to note that JrPRP represents a crude fraction, and its detailed structural features remain to be fully elucidated. Further investigations using advanced analytical techniques, such as NMR spectroscopy, molecular weight determination, and LC–MS analysis, are required to confirm its structural organization and better understand structure–function relationships. In addition, in vivo studies and detailed mechanistic investigations will be necessary to validate its potential for biomedical and functional applications.

## Figures and Tables

**Table 1:** Chemical composition and monosaccharide composition of JrPRP.

Biochemical Composition (%)					
Total sugar		Reducing sugar		Proteins	Lipids
63.2 ± 0.4		40.3 ± 0.1		34.1 ± 0.1	2.6 ± 0.2
**Saccharide Composition (%)**					
Major carbohydrate fraction (unassigned peak)	Sucrose	Glucose	Galactose	Mannose	Arabinose
92.2 ± 1.2	2.1 ± 0.1	2.1 ± 0.1	1.5 ± 0.3	0.7 ± 0.1	1.3 ± 0.2

**Table 2:** Total antioxidant activities of JrPRP polysaccharide.

IC_50_ (DPPH)	IC_50_ (ABTS)	IC_50_ (FRAP)	IC_50_ (Chelating Power)
405 ± 1.8 µg/mL	225 ± 3.5 µg/mL	338.3 ± 2.1 µg/mL	229.8 ± 1.7 µg/mL

**Table 3:** MIC, MBC and CMB/MIC ratio of JrPRP.

Bacterial Strains	JrPRP		
	MIC (mg/mL)	MBC (mg/mL)	MBC/MIC
*Bacillus cereus*	9	10	1.1
*Klebsiella pneumonia*	6	7	1.1
*Staphylococcus aureus*	5	7	1.4
*Escherichia coli*	4	6	1.5
*Enterobacter* sp.	4	6	1.5
*Pseudomonas aeruginosa*	2	3	1.5

**Table 4:** Dose-Dependent Effect of *Juglans regia* Root Bark Polysaccharides on Coagulation Parameters (PT, PT ratio, and INR).

JrPRP (µL/mL)	PT (s)	PT Ratio	INR (ISI = 1.3)
Control	12.5	1.000	1.000
1	14.8	1.184	≈1.24
1.5	16.3	1.304	≈1.41
2	17.3	1.384	≈1.52
2.5	19.1	1.528	≈1.70

## Data Availability

The original contributions presented in this study are included in the article. Further inquiries can be directed to the corresponding author.
